# Suicidal attempt with eliglustat overdose

**DOI:** 10.1002/jmd2.12341

**Published:** 2022-10-04

**Authors:** Johannes Nadler, Maren Hermanns‐Clausen, Karin Dilger

**Affiliations:** ^1^ Department of General Pediatrics, Adolescent Medicine and Neonatology, Center for Pediatrics Medical Center – University of Freiburg, Faculty of Medicine, University of Freiburg Freiburg Germany

**Keywords:** eliglustat, Gaucher disease, overdose, poisoning, safety, suicide attempt

## Abstract

Eliglustat is an orphan medicine used for long‐term treatment of Gaucher disease type 1 (GD1) in adults. GD1 is a genetic condition, in which glucosylceramide builds up in the body, typically in liver, spleen, and bone. Clinical signs and symptoms of the disease are anemia, tiredness, easy bruising, hepatosplenomegaly, bone pain, and fractures. Eliglustat works by blocking glucosylceramide synthase (substrate reduction therapy). This medicine is subject to additional safety monitoring by regulatory authorities in the European Union. Scientific literature on eliglustat overdose is not available. We herein describe successful treatment of a suicidal attempt with massive eliglustat overdose. A 29‐year‐old female with GD1, a poor metabolizer of cytochrome P450 2D6 on a recommended daily dose of 84 mg of eliglustat, had taken 94 capsules of eliglustat (84 mg per capsule). One hour after ingestion of almost 8 g of eliglustat, the patient suffered from somnolence, severe bradycardia (37 bpm), and hypotension (systolic blood pressure of 70 mm Hg). After intravenous administration of atropine (1 mg) and cafedrine/theoadrenaline (100 mg/5 mg) by the called emergency physician, the patient resolved gradually. She remained 24 h with stable hemodynamics at a nearby intensive care unit. During continuous ECG monitoring, increased frequency of supraventricular ectopic activity and a first‐degree atrioventricular block were observed. To our knowledge, this is the first case report on a suicidal attempt with eliglustat.

**Synopsis**

Physicians must be aware of immediately serious cardiovascular reactions (bradycardia, low blood pressure, and irregular heartbeat) following a large overdose of eliglustat.

## 1 INTRODUCTION

Eliglustat is an orphan medicine used for long‐term treatment of Gaucher disease type 1 (GD1) in adults.^1^ GD1 is an autosomal recessive lysosomal storage disease that results from deficiency of the lysosomal enzyme β‐glucosidase (also known as glucocerebrosidase).^2^ β‐Glucosidase is the rate‐limiting enzyme in the catabolism of complex glycosphingolipids, catalyzing the hydrolysis of glucosylceramide to glucose and ceramide. In GD1, glucosylceramide builds up in the body, typically in liver, spleen, and bone.^3^ In the general population prevalence of Gaucher disease is one in 50 000 to 100 000 individuals.^4^, ^5^ GD1, the non‐neuronopathic variant of Gaucher disease, accounts for almost 95% of cases among Caucasians.^6^ Infiltration of the reticuloendothelial system by Gaucher cells, which are lipid‐engorged macrophages with a characteristic crinkled‐paper cytoplasmic appearance, is central to pathophysiology of the disease.^7^ Clinical signs and symptoms of GD1 are anemia, tiredness, easy bruising due to thrombocytopenia, hepatosplenomegaly, bone pain, and pathological fractures.^3^, ^8^ Eliglustat is a new substrate reduction therapy that inhibits glucosylceramide synthase, thereby reducing the synthesis of glucosylceramide.^9^ This medicine is subject to additional safety monitoring by regulatory authorities in the European Union.^10^ Scientific literature on eliglustat overdose is not available. We herein describe successful treatment of a suicidal attempt with a megadose of eliglustat.

## 2 CASE PRESENTATION

Our patient is a 29‐year‐old female (height 164 cm, body weight 103 kg, body mass index 38 kg/m^2^) with GD1. GD1 was diagnosed 10 years ago by molecular testing of the *GBA1* gene revealing two pathogenic mutations (c.475C>T [p.Arg159Trp] in exon 5; c.1226A>G [p.Asn409Ser] in exon 9). On examination and investigation at the emergency department, she showed the following signs of GD1: splenomegaly, anemia (hemoglobin level 11.3 g/dl, red blood cell count 4.01 million cells/μl, and hematocrit level 34.4%), and thrombocytopenia (148 000/nl). The patient was on long‐term substrate reduction monotherapy with oral eliglustat (daily dose 84 mg) for 2 years. This dosage regimen is based on her cytochrome P450 2D6 genotype (poor metabolizer, PM).^1^ The patient did not take any concomitant medication. In February 2021, in a suicidal attempt, she ingested all at once 94 capsules of eliglustat (84 mg of eliglustat per capsule) which is a total of almost 8 g of eliglustat. The patient herself called the ambulance within the first hour after ingestion of the medication. Upon arrival of the emergency physician, she was already somnolent and hemodynamically unstable. The patient showed severe bradycardia (37 bpm) and hypotension (70/50 mmHg). The emergency physician promptly administered atropine (1 mg) and also cafedrine/theoadrenaline (100 mg/5 mg) into a forearm vein. When she was in a stable condition, the patient was transferred to the nearby emergency unit. Upon arrival at hospital, heart rate and blood pressure were no more below normal (heart rate 80 bpm and blood pressure 100/75 mmHg), and the patient remained in a stable condition. No respiratory insufficiency was detected (oxygen saturation 94% and respiratory rate 10/min). Neurological examination revealed mild somnolence with lack of concentration. A complete blood analysis showed normal serum electrolyte levels, normal liver and kidney function, and normal blood glucose levels. A broad toxicology screening in urine (methamphetamine, cocaine, tetrahydrocannabinol, ecstasy, methadone, morphine/heroine, benzodiazepines, tricyclic antidepressants, barbiturate, amphetamines, and buprenorphine) was negative. During 24 h of continuous electrocardiographic monitoring at the intensive care unit an increased frequency of supraventricular ectopic activity and mild first‐degree atrioventricular block (PR interval: 206 ms) were observed. The patient improved gradually by supportive care (fluid therapy). After 24 h, the reactions had resolved completely and the patient was transferred to a psychiatric facility for follow‐up (suspected undiagnosed schizophrenia). She has reported to a clinician on auditory hallucinations as cause of her suicidal attempt: commanding voices had told her to take the drugs.

## 3 DISCUSSION

To our knowledge, this is the first case report on a suicidal attempt with eliglustat. In our patient (CYP2D6 PM), intake of a megadose of eliglustat resulted in serious cardiovascular symptoms together with somnolence within the first hour of absorption.

The indication for eliglustat is long‐term treatment of adult patients with GD1.^10^ The outcomes of phase 2 and phase 3 trials show that eliglustat is effective in reversing cardinal features of GD1 (spleen volume, hemoglobin level, and platelet count).^11^, ^12^, ^13^, ^14^ The recommended oral dosage is 84 mg eliglustat once daily in CYP2D6 poor metabolizers (PM) and 84 mg twice daily in CYP2D6 extensive or intermediate metabolizers.^1^ After oral administration, maximum eliglustat plasma concentrations are observed after 2 h.^15^ Absolute bioavailability of eliglustat is below 5%.^16^ The plasma elimination half‐life of eliglustat is 6 h in CYP2D6 intermediate metabolizers and wild‐type subjects and increased up to 10 h in CYP2D6 PM.^16^

At therapeutic dosage, the majority of side effects of eliglustat are mild and short‐lived.^17^ Common reactions are gastrointestinal disorders.^10^ The most frequently reported treatment‐related serious adverse event occurring during eliglustat treatment in clinical trials was syncope (incidence 0.76%) with all of these events being associated with pre‐disposing risk factors and being of vasovagal nature.^16^ Patients with heart disease or heart rhythm problems were excluded from clinical trials, since there is a potential risk of an effect on the electrical activity of the heart when levels of eliglustat in the blood are very high.^18^ During single dose escalation in a phase 1 trial, dose‐limiting toxicity was met at 30 mg/kg eliglustat in a 30‐year‐old male due to hypotension, bradycardia, nausea, and vomiting.^15^ For comparison, our patient with GD1 showed serious cardiovascular reactions after a single dose of about 76 mg/kg eliglustat.

In the European Union, medicines that are being monitored particularly closely by regulatory authorities are labeled with a black inverted triangle in the product information. Eliglustat is subject to additional safety monitoring.^10^ An increased frequency of supraventricular ectopic activity and first‐degree atrioventricular block as observed in our patient is unexpected (reactions not given in the section on undesirable effects of the product information).^10^ A small number of study patients treated with eliglustat in clinical trials experienced mild events of irregular or abnormal heartbeat. In most cases, they were considered not related to treatment with eliglustat.^18^ However, in phase 2, one study patient showed a short run of asymptomatic non‐sustained ventricular tachycardia possibly related to intake of eliglustat.^11^ In the phase 3 trials, there were two treatment‐related events of second‐degree atrioventricular block in one patient considered serious, and one treatment‐related event of arrhythmia resulting in withdrawal of the patient.^13^, ^14^

Our patient with a large overdose of eliglustat (94‐fold of recommended dose) presented with severe bradycardia and hypotension, which are symptoms of parasympathetic stimulation, together with impaired consciousness. Immediate administration of intravenous atropine together with a 20:1 combination of cafedrine:theodrenaline, widely used in Germany for the treatment of hypotensive states in emergency medicine, restored hemodynamic stability. Cafedrine is a chemical linkage of norephedrine and theophylline; theodrenaline is a linkage of nordadrenaline and theophylline. Cafedrine/theodrenaline leads to a rapid and long‐lasting increase in mean arterial pressure characterized by increased cardiac preload, stroke volume, and cardiac output.^19^ Systemic vascular resistance and heart rate remain mostly unchanged.^19^ Bradycardia can be due to sinus, atrial, or junctional bradycardia or to a problem with the conduction system (e.g., an atrioventricular block). Bradycardia symptoms may include syncope or dizziness. In the acute setting, a critically poisoned patient with bradyarrhythmia should be treated with atropine.^20^

Eliglustat is predominantly metabolized by CYP2D6 and to a lesser extent by CYP3A4.^16^ Concomitant medicines inhibiting CYP2D6 or CYP3A4 activity may increase significantly eliglustat plasma concentrations. Therefore, physicians should also keep in mind that patients may be at risk because of drug interaction.

In conclusion, physicians must be aware of immediately serious cardiovascular reactions (bradycardia, low blood pressure, and irregular heartbeat) following intake of a large overdose of eliglustat. We recommend treatment with atropine and a vasopressor in case of emergency.

## AUTHOR CONTRIBUTIONS

Johannes Nadler: Collecting data, interpretation of the data, literature search, preparing an early draft of the manuscript. Maren Hermanns‐Clausen: Planning, analysis and interpretation of the data. Karin Dilger: Interpretation of the data, literature search, preparing the manuscript and writing the final version. All authors read and approved the final manuscript.

## CONFLICT OF INTEREST

The authors declare no conflicts of interest.

## PATIENT CONSENT

A signed patient consent was obtained prior to writing of the case report. Proof that a signed patient consent was obtained is available upon request.

## Data Availability

The data supporting the results reported in the article are available from the corresponding author upon reasonable request.
